# Assessing enablement in clinical practice: a systematic review of available instruments

**DOI:** 10.1111/j.1365-2753.2009.01332.x

**Published:** 2010-12

**Authors:** Catherine Hudon, Denise St-Cyr Tribble, France Légaré, Gina Bravo, Martin Fortin, José Almirall

**Affiliations:** 1 Associate Professor, Department of Family Medicine, Université de SherbrookeSherbrooke, Québec, Canada; 2 Full Professor, School of Nursing Science, Université de SherbrookeSherbrooke, Québec, Canada; 3 Associate Professor, Department of Family Medicine, Université LavalQuébec, Québec, Canada; 4 Full Professor, Department of Community Health Sciences, Université de SherbrookeSherbrooke, Québec, Canada; 5 Full Professor, Department of Family Medicine, Université de SherbrookeSherbrooke, Québec, Canada; 6 Research Assistant, Centre de santé et de services sociaux de ChicoutimiChicoutimi, Québec, Canada

**Keywords:** empowerment, enablement, instruments, professional practice, questionnaires, systematic review

## Abstract

**Rationale, aims and objectives:**

Enablement is an intervention by which the health care provider recognizes, promotes and enhances patients' ability to control their health and life. An abundant health literature suggests that enablement is associated with good outcomes. In this review, we aimed at identifying and comparing instruments that assess enablement in the health care context.

**Method:**

We conducted a systematic literature review using Medline, Embase, Cochrane, Cinahl and PsycINFO databases, 1980 through March 2009, with specific search strategy for each database. Citations were included if they reported: (1) development and/or validation of an instrument; (2) evaluation of enablement in a health care context; and (3) quantitative results following administration of the instrument. The quality of each main retained citation was assessed using a modified version of the Standards for Reporting of Diagnostic Accuracy.

**Results:**

Of 3135 citations identified, 53 were retrieved for detailed evaluation. Four articles were included. Two instruments were found: the Patient Empowerment Scale (PES) and the Empowering Speech Practices Scale (ESPS). Both instruments assessed enablement in hospital setting, one from the inpatient's perspective (PES) and the other from both perspectives (ESPS).

**Conclusion:**

Two instruments assess enablement in hospital setting. No instrument is currently available to assess enablement in an ambulatory care context.

## Introduction

Enablement is an intervention by which the health care provider recognizes, promotes and enhances people's ability to control their health and life [1–4]. St-Cyr Tribble *et al.* propose, in accordance with recent reviews on the topic [1–4], a model of enablement regrouping the following dimensions: contributing to the therapeutic relationship, building on the person's point of view, facilitating the learning experience, encouraging and supporting the decision-making process, building on the person's strengths and helping to broaden the person's possibilities [3].

Enablement is in continuity with the patient-centred model [5,6] because it shares many dimensions with this last model (contributing to the therapeutic relationship, building on the person's point of view, encouraging and supporting the decision-making process and facilitating the learning experience). The term ‘empowerment’ is often used to talk about enablement [7–10]. However, this term (empowerment) may be confusing because it can also represent the patient's outcome after the enablement intervention [2,4,11].

Abundant health literature suggests that strategies based on enablement are associated with good outcomes [12], which include a more effective decision-making process, a better management of disease complications and adoption of healthier behaviours [13]. However, despite these potential relevant outcomes, the World Health Organization stressed the lack of patients' enablement by health care providers in the long-term care of chronic diseases [12]. A primary step in developing and evaluating strategies to improve enablement is to have a reliable and valid measure of enablement in the health care context. As our capacity to measure it grows, we will be in a better position to estimate the real effects of enablement on patients' health, and more broadly, on the health care system [14]. We could also be able to design effective strategies to improve enablement.

In this study, we aimed at identifying and comparing instruments used to assess enablement in the health care context.

## Methods

### Search strategy and articles selection

Covering all available years from 1980 to March 2009, we conducted an electronic literature search of the following databases: Medline (1980–), Embase (1980–), Cochrane (1991–), Cinahl (1982–) and PsycINFO (1980–), without any language restriction. An information specialist was consulted to help develop, update and run specific strategies for each databases. The following MeSH terms and keywords were used: ‘Power (psychology)’, ‘Empowerment’, ‘Empowering’, ‘Enablement’, ‘Questionnaires’, ‘Process assessment’, ‘Quality assessment’, ‘Psychometrics’, ‘Scale’, ‘Instrument construction’, ‘Instrument validation’, ‘Instrument scaling’, ‘Validation studies’, ‘Nursing assessment’, ‘Reliability’, ‘Validity’, ‘Test validity’, ‘Test reliability’, ‘Test construction’, ‘Factor analysis’, ‘Rating scale’, ‘Instrument’, ‘Measurement’, ‘Assessment’, ‘Tool’, ‘Health Care Services’, ‘Continuum of Care’, ‘Long Term Care’, ‘Mental Health Services’, ‘Palliative Care’, ‘Primary Health Care’. We also examined additional relevant articles that potentially included an eligible instrument from the reference lists of the collected articles (hand searching).

All searches were transferred to a reference database (Refworks). Refworks gave the opportunity to group similar (not necessarily identical) citations. Similar citations were then checked to remove manually the identical ones (citations indexed on more than one database). The titles and abstracts were read one by one (CH). We excluded the citation at this stage if it was obvious that it did not satisfy our inclusion criteria. We retained other citations for complete reading. Two authors (CH and JA) independently appraised the full text of identified retained papers to identify potentially eligible articles. Discrepancies between the two reviewers were resolved by consensus. First authors of included studies were contacted to identify uncovered articles.

### Inclusion and exclusion criteria

Citations were retained if they satisfied all of the following criteria: (1) development and/or validation of an instrument; (2) evaluation of enablement (often called ‘empowerment’) defined as an intervention by which the health care provider recognizes, promotes and enhances patients' abilities to control their health and lives; and (3) quantitative results following administration of the instrument. We did not search for articles measuring only a sub-concept or a dimension of enablement (patient-centred care, shared decision making, participation and information, etc.). Articles including only a subscale on enablement were not retained.

### Assessment of study quality

We assessed the quality of the first article published (considered as the main article) for each instruments selected, using a modified version of the Standards for Reporting of Diagnostic Accuracy (STARD) [15–17]. The STARD is an outgrowth of the Consolidated Standards of Reporting Trials initiative [18], adopted by many of the leading biomedical journals and journals in psychology [19]. The STARD has already been successfully used in a similar systematic review by a member of our team (F. L.) [20]. From the initial STARD that contained 25 items [15–17], 15 items were kept and judged appropriate to evaluate questionnaires. Using this modified scale (15 items), two researchers (CH and JA) independently determined a global quality score for each article. The scores were then compared and a consensus was reached.

### Data extraction

The following data were extracted for each instrument included: [21] name of the instrument as given by the original author, first author of the instrument and year of publication, discipline of the first author, main purpose (measurement aim, clinical domain and context of use envisioned by author, including the person who completed the instrument), description of the instrument (number of dimensions and items), response scale, development procedures, conceptual/theoretical foundation and psychometric properties. Special attention was given to face and content validity [19]. Face validity indicates whether the instrument appears to be assessing the construct of interest. Content validity is the extent to which all dimensions of the measured concept are represented in the instrument [19]. Data extraction was completed by two members of the team (CH and JA) and disagreements were resolved by consensus.

## Results

### Articles included

**Figure 1:**
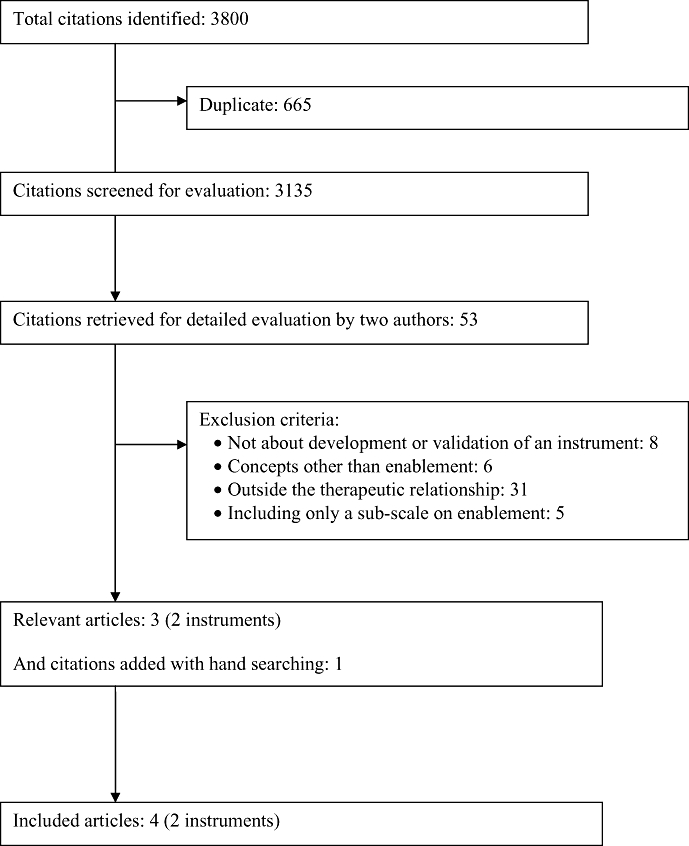
Number of citations identified through the stages of the systematic review.

Figure 1 shows the number of citations found at each stage of the selection process. The search strategies identified 3800 citations, from which 3135 were kept after removing duplicates. Fifty-three were read completely and evaluated. Among these articles, eight were not about the development or validation of an instrument [22–29]; six were about concepts other than enablement or empowerment [30–35], 31 did not measure a professional intervention [36–66] and five included only a subscale on enablement [67–71]. Hand searching and correspondence with authors added another relevant article [72].

Four articles covering two instruments were included in this review. The identified instruments were: the Patient Empowerment Scale (PES) [72,73] and the Empowering Speech Practices Scale (ESPS) [74,75].

### Quality assessment

Overall, the quality of the studies was fair: 8 and 9 out of 15 (Table 1).

**Table 1:** Quality assessment of the main studies included, based on the modified version of Standards for Reporting of Diagnostic Accuracy

Section and topic	Item	Patient Empowerment Scale [72,73]	Empowering Speech Practices Scale [74,75]
Title/abstract	Identify the article as a study concerning a measuring instrument.	+	+
Introduction	State the research question or study aims, like developing or validating a measuring instrument.	+	+
Methods Participants	Describe the study population: The inclusion and exclusion criteria, setting and locations where the data were collected.	+	0
	Describe the method of recruitment of the participants.	+	+
	Describe participant sampling: Was the study population a consecutive series of participants? If not, specify how participants were further selected.	0	+
Test methods	Describe technical specifications of material and methods involved, including how and when measurements were taken, and/or cite references for measuring instrument.	+	+
	Describe relevant information for the readers concerning the measuring instrument (scale available in the text).	+	0
Statistical methods	Describe methods for calculating or comparing measures of reliability, validity and the statistical methods used to quantify uncertainty (e.g. 95% confidence intervals).	0	0
Results Participants	Report when study was done, including beginning and ending dates of recruitment.	0	+
	Report demographic characteristics of the study population (e.g. age, sex, employment, recruitment centres).	+	+
	Report the number of participants satisfying the criteria for inclusion (a flow diagram is strongly recommended).	0	0
Test results	Report distribution of severity of the situation being assessed.	0	0
Estimates	Report estimates of accuracy and measures of statistical uncertainty (e.g. 95% confidence intervals).	0	0
	Report how indeterminate results, missing responses and outliers on the measuring instrument were handled.	0	+
Discussion	Discuss the clinical applicability of the study findings.	+	+
Total score		8/15	9/15

### Characteristics of instruments

Tables 2 and 3 summarize both included instruments. Both instruments (PES and ESPS) were based on conceptual frameworks developed from studies conducted by the same authors (as the instrument development).

**Table 2:** Characteristics of the two instruments included

Name of the instrument (First author, year of publication)	Origin of first author, country	Main purpose (Measurement aim, clinical domain and context of use envisioned by author)	Description (number of dimensions and items)	Response scale
Patient Empowerment Scale (Faulkner, 2001) [72,73]	Department of Community Ageing Rehabilitation Education and Research, UK	To assess inpatients' enablement in hospital environments.	3 dimensions in the empowerment subscale (20 items): promoting patient independence, awareness of patients' needs and promoting information exchange	3-point Likert
		Completed by the patient after at least 3 days of hospitalization.		
			3 dimensions in the disempowerment subscale (20 items): impeding patient collaboration in care planning, domination and indifference to patients' needs.	
Empowering Speech Practices Scale (Kettunen, 2006) [74,75]	Research Center for Health Promotion, Finland	To assess enablement of dyadic counselling in hospital setting.	2 dimensions (44 items): professionally led conversation and patient's requests for additional clarification.	3-point Likert
		Completed by the inpatient and the nurse (by way of the same statements, parallel questionnaires) after the counselling encounter.		

**Table 3:** Development and psychometric properties of the two instruments included

Instrument	Origins and development	Conceptual framework	Validity	Reliability
Patient Empowerment Scale [72,73]	38 registered nurses were asked to nominate empowering and disempowering acts relevant to interactions between staff and elderly patients. Further acts were obtained following a literature review.	Constructed on the basis of a conceptual model of empowerment and disempowerment derived from the literature and empirical results.	Face validity: Patients and nurses were consulted.	Internal consistency: Cronbach alpha from 0.75 to 0.87 for the empowerment subscale and 0.65 to 0.88 for the disempowerment subscale.
	The resulting lists of 98 acts for each disposition were hypothetically judged by older hospitalized people as to the extent they would be either ‘control giving’ (empowering acts, *n* = 20) or ‘control taking’ (disempowering acts, *n* = 20) if personally experienced. The 20 highest scoring acts in each category were incorporated into the Patient Empowerment Scale.		Content validity: The scale is coherent with the conceptual model developed, but does not take into account all the literature on empowerment.	
	The instrument was tested on 102 elderly (65 years or older) patients from three hospitals (inpatients for at least 3 days) in Central England (acute medicine, surgery and elderly care rehabilitation).		Construct validity: Factor analysis (not detailed in the article).	
Empowering Speech Practices Scale [74,75]	The first stage was a conversation of counselling encounters.	Constructed on the basis of a conceptual model of empowerment derived from a case study by the authors.	Face validity: Patients and nurses were consulted.	Internal consistency: Cronbach alpha for global score: 0.88 and for subscales: 0.52 to 0.83.
	At the second stage, the study was used to design a questionnaire containing 65 statements.		Content validity: This scale simultaneously includes items related to the enablement process and outcomes	
	At the third stage, interviews were conducted where five patients and five nurses assessed their videotaped counselling sessions by means of the instrument. The statements were reduced to 58.			
	At the fourth stage, the revised instrument was tested by four patients on a hospital ward.			
	Finally, 127 counselling situations involving adult patients (56 women and 54 men; mean age = 54.9 years) were evaluated with nurses (mean age = 41.7; average experience = 13.8) on 17 wards and polyclinics providing care to adult patients in Finland. This left a total of 44 statements.		Construct validity: Factor analysis confirms two factors accounting for 59% of the variance. Average loading for professionally led conversation: 0.80 and patient's requests for additional clarification: 0.84.	

The PES evaluated patient's perception, whereas ESPS was completed by both patient and nurse with parallel questionnaires, thus representing both perceptions of the consultation. Both instruments targeted inpatients. Both instruments used a 3-point Likert response scale. The PES contained 40 items and the ESPS contained 44 items.

The PES was studied among specific patient populations (elderly inpatients), while ESPS was validated among a more diverse group of patients. Both studies involved nurses and were conducted in Europe.

## Discussion

To the best of our knowledge, this is the first review to identify and compare instruments used to measure enablement in a health care context. Two instruments were identified. The PES is a 40-item instrument to assess inpatient's perception of enablement. The ESPS, a 44-item instrument, assesses enablement in hospital setting, from the patients' and nurses' perspectives.

Both instruments were based on conceptual frameworks developed from studies conducted by the original authors of the instrument. Basing instrument development on conceptual framework may increase the content validity of the instrument [76–78]. However, it is relevant to start with a critical review of the literature to have a more valid conceptual model [79,80]. As suggested by Streiner and Norman [19], recent reviews and empirical research [1–4] could be used in further attempts to improve these instruments (PES and ESPS) or elaborate new ones.

The enablement model [1,3,4] and the patient-centred model [5,81] share many common dimensions (contributing to the therapeutic relationship, building on the person's point of view, encouraging and supporting the decision-making process and facilitating the learning experience), raising the question ‘Is it the same concept?’ Our view is that the enablement process is necessarily patient-centred but a patient-centred intervention does not always correspond to an enablement intervention. Indeed, building on the person's strengths is central to the enablement model [1,82–85].

Another question that could be raised is ‘How is the enablement concept positioned among other concepts such as shared decision making, patient participation . . .’ As stressed in our enablement model (see *Introduction*), these last concepts could be considered as sub-concepts of enablement. Our hypothesis is that improving many aspects of the interaction between the patient and the health care provider (referring to the dimensions of our enablement model) is maybe more effective than improving only one or two aspects.

The PES evaluates patient's perception, while ESPS considers patient and professional's points of view. Some authors argued that both points of view have to be taken into account to evaluate professional's practices, because interaction of these points of view is determinant in the intervention process [11,86]. Other studies demonstrated stronger relationship between patient's perceptions and various outcomes. For example, in one study, perceived empowering care by patients was the most important predictor of their quality of life [87]. Stewart *et* *al*. evaluated patient-centredness, a concept regrouping many dimensions of enablement. They demonstrated that the patient's perception was a better predictor of certain outcomes at the end of a 2-month period than that of a trained person scoring an instrument from the audiotape of the encounter. One of their conclusions was that the patient outcomes will be affected only when the doctor's patient-centeredness reaches a level that the patient notices [5]. This conclusion may be true for enablement as well. Further studies are needed to evaluate this hypothesis.

The existing instruments are cross-sectional measures to assess enablement (PES and ESPS). It is appropriate when considering patients provided with medical care in an inpatient context. However, in an ambulatory care setting, because the interaction between a patient and a health care provider (his or her family doctor for example) may evolve over time, the enablement intervention may develop on a longitudinal basis [88,89]. Therefore, one has to wonder, before using these instruments in such a context, whether they are appropriate to measure an inherently longitudinal process. This perspective must be kept in mind while developing or improving instruments to assess enablement in an ambulatory care context.

### Limitation of the study

One of the main limitations of a review is its inability to include all of the relevant literature and unpublished material. We acknowledge that some eligible articles may have been missed during the search stage. However, our search strategy was adapted for different databases, was developed with an information specialist and favoured an exhaustive literature review. Our hand search and correspondence with experts were other ways to help us identify uncovered articles. We did not include articles in which enablement was part of a larger concept, such as quality of care, because of the risk of missing such subscales. Scales measuring sub-concepts of enablement (shared decision making for example) or measuring enablement as a sub-concept of another construct could, however, be used in a pool of items in further instrument development.

### Conclusion

Two instruments (PES and ESPS) were identified that measure enablement in the health care context. Both instruments assess enablement in hospital setting, one from the patient's perspective (PES) and the other from both the patient's and the nurse's perspective (ESPS). To date, there is no tool available to the clinicians or researchers to assess enablement in an ambulatory care context.
